# Déterminants de l'utilisation des services de santé en République du Congo: cas des ménages du District Sanitaire de Moungali

**DOI:** 10.11604/pamj.2025.50.31.37841

**Published:** 2025-01-22

**Authors:** Arkadit Jeandria Nkodia, Steven Richy Missongo, Jesse Saint Saba Antaon, Ceverly Hortège Dieudonné Milandou, Prisca Doriane Ngnikam Tienkeu, Bermeland Ewuinh Tsiobinda

**Affiliations:** 1 Association Congolaise pour la Santé Publique et Communautaire, Brazzaville, République du Congo,; 2 Faculté de Médecine et des Sciences Biomédicales, Université de Yaoundé I, Yaoundé, Cameroun,; 3 District Sanitaire de Moungali, Brazzaville, République du Congo,; 4 Environnement Recherche Action, Yaoundé, Cameroun

**Keywords:** Déterminants, utilisation, services de santé, ménages, Congo, Determinants, utilization, health services, households, Congo

## Abstract

**Introduction:**

l'utilisation des services de santé reste un défi majeur pour atteindre la couverture sanitaire universelle. Cependant, en République du Congo, peu d'études mettent en évidence les déterminants de leur utilisation. L´objectif de notre étude était d´estimer le taux d´utilisation des services de santé à Brazzaville et d'identifier ses déterminants: le cas des ménages du District Sanitaire de Moungali en 2021.

**Méthodes:**

nous avons réalisé une étude transversale à visée analytique auprès de 800 chefs de ménage du District Sanitaire de Moungali durant le mois de septembre 2021. Les données ont été collectées par la méthode CAPI (*Computer Assisted Personal Interviewing*) à l'aide de Kobo Collect et analysées avec le programme R version 4.2.0. La régression logistique binaire a permis d´identifier les déterminants.

**Résultats:**

le taux d'utilisation des services de santé a été de 27,62% IC à 95% [24,62-30,83]. Les déterminants de l´utilisation des services de santé ont été: un revenu supérieur au salaire minimum interprofessionnel garanti (SMIG), ORA (Odds Ratio Ajusté) =2,12 IC à 95% [1,30-3,48], p=0,003); le coût des soins faible (ORA=1,73 IC à 95% [1,11-2,7], p=0,015) et le délai d´attente court dans les services de santé (ORA=1,90 IC à95% [1,21-2,97], p=0,001).

**Conclusion:**

le taux d'utilisation des services de santé est faible et constitue encore un défi majeur pour l’atteinte des cibles de santé des ODD 3 et la couverture sanitaire universelle. Des mécanismes d´assurance maladie et de solidarité nationale doivent être mis en place pour améliorer l'accès aux soins et services de santé. Ces actions nécessitent une intervention multisectorielle.

## Abstract (en)

**Introduction:**

health service utilization remains a major challenge for achieving universal health coverage. However, in the Republic of Congo, few studies highlight the determinants of health service use. The purpose of our study is to estimate the health service utilization rate in Brazzaville and identify its determinants, focusing on households in the Mo**ungali Health District in 2021**.

**Methods:**

we conducted an analytical cross-sectional study involving 800 household heads in the Moungali Health District during the month of September 2021. Data were collected using the CAPI (Computer Assisted Personal Interviewing) method through Kobo Collect and analyzed with R software version 4.2.0. Binary logistic regression was **used to identify determinants**.

**Results:**

the health service utilization rate was 27.62%, 95% CI [24.62-30.83]. The determinants of health service utilization were: income above the minimum wage (SMIG - Guaranteed Minimum Interprofessional Salary), AOR (Adjusted Odds Ratio) =2.12 95% CI [1.30-3.48], p=0.003; low healthcare costs (AOR=1.73 95% CI [1.11-2.7], p=0.015) and short waiting time in health services (AOR=1.90 **95% CI [1, 21-2.97], p=0.001)**.

**Conclusion:**

the health service utilization rate is low, remaining a significant challenge for achieving SDG 3 health targets and universal health coverage. Health insurance mechanisms and national solidarity initiatives should be implemented to improve access to healthcare and health services. These actions require multisectoral intervention.

## Introduction

Selon un rapport conjoint de la Banque Mondiale et de l´Organisation mondiale de la Santé (OMS), au moins une moitié de la population mondiale n´a pas accès aux services de santé essentiels [1]. L´accès à tous aux services de santé dont ils ont besoin sans faire face à des difficultés financières est essentiel pour améliorer le bien-être de la population d´un pays [2]. Les pays en développement montrent la caricature des insuffisances d´une organisation des services de santé fondée sur une logique biomédicale [3]. L'Afrique est le continent où la situation sanitaire et sociale est la plus préoccupante [4]. Cependant, en République du Congo, peu de données scientifiques existent sur les déterminants de l´utilisation des services de santé, que ce soit en milieu urbain ou en milieu rural. La connaissance scientifique de ces déterminants contribuera à favoriser une amélioration progressive de la qualité de l´offre par les personnels de santé et son utilisation par les ménages. Dans ce contexte nous nous sommes posé la question de savoir: quels sont les déterminants de l´utilisation des services de santé à Brazzaville? le cas des ménages du District Sanitaire de Moungali en 2021. Malgré la rareté des données et au regard de la littérature tirée des autres pays, nous estimons que la proportion des ménages utilisant les services de santé en cas de maladie est faible. Les caractéristiques sociodémographiques et les conditions de vie des ménages influencent sur l'utilisation des services de santé. Les objectifs spécifiques de notre étude étaient: i) estimer le taux d´utilisation des services de santé, ii) identifier les déterminants de l´utilisation des services de santé par les ménages.

## Méthodes

### Type d´étude et population

Nous avons réalisé une étude transversale à visée analytique auprès des chefs de ménages du District Sanitaire de Moungali durant le mois de septembre 2021. Les chefs de ménages étaient appuyés par leurs conjointes pour répondre au formulaire dans le cas du couple.

### Critères d'inclusion et critères d'exclusion

***Inclusion:*** les chefs de ménages des parcelles tirées au sort: ayant vécu au moins une année dans le District Sanitaire de Moungali; présents au moment de l´enquête et ayant donné leur consentement pour participer à l'enquête.

***Exclusion:*** les chefs de ménages habitant dans les parcelles tirées au sort: absents au moment de l´enquête, présents au moment de l´enquête mais n´ayant pas donné leur consentement à participer à l'enquête et n´ayant pas vécu au moins une année dans le District Sanitaire de Moungali.

### Type d´échantillonnage

Un échantillonnage de type probabiliste par sondage à deux degré (sondage à plusieurs degrés) a été réalisé. Nous avons procédé par un sondage aléatoire simple des zones de dénombrement (ZD) au 1^er^ degré. Quatre (4) ZD ont été tirées au sort dans chaque quartier. Au total, quarante (40) ZD ont été tirées au sort dans l´ensemble de neuf (09) quartiers du District Sanitaire de Moungali (Arrondissement de Moungali). Ensuite, un choix aléatoire de 20 parcelles a été réalisé dans chaque ZD (grappe) pour atteindre un échantillon total de 800 parcelles. Un seul ménage a été interrogé dans chaque parcelle, soit un total de 800 ménages. Les enquêteurs procédaient à une subdivision de la ZD en quatre parties puis se plaçaient au centre de la ZD. La direction à prendre était déterminée au hasard à partir de l'orientation indiquée par le stylo tournoyé ou pivoté au sol, la direction qu´indiquait le stylo était considérée comme celle de la ZD éligible. Le choix de la première parcelle se faisait à l´aide de la table de nombre aléatoire en choisissant de manière contigu un nombre compris entre 1 et 20 après avoir affecté aléatoirement à chaque parcelle un numéro unique. Au cas où on avait plusieurs portes dans une même parcelle, le choix de la porte du ménage à entretenir se faisait au hasard en se servant du stylo lancé en l´air tournoyé ou pivoté au sol. En cas d´absence du ménage ou de non-continuité de la ruelle ou avenue (impasse), un choix de la direction au hasard se faisait en se servant du stylo. Cette procédure était exécutée jusqu´à l´atteinte de la taille escomptée pour chaque grappe.

### Taille de l´échantillon

La taille minimum de notre échantillon a été calculée à partir de la formule de Daniel Schwartz:


$$n\geq z^{2}\frac{p.q}{i^{2}}k$$

n= taille minimale de notre échantillon; z= paramètre lié au risque d´erreur, fixé à 0,05 et correspondant à 1,96 dans la table des écarts réduits; i= précision absolue souhaitée (i=0,05); P= proportion de l´utilisation services de santé; q=1-P, k=effet de grappe=2. En absence d´une proportion préalable en République du Congo, nous avons choisi la proportion d´une étude réalisée en République Démocratique du Congo en 2013, dans la province du Katanga qui rapportait une proportion de l'utilisation des services curatifs de 47% [5]. On a obtenu: z=1,96; p=0,47; q=0,53; i=0,05

n = (1,96)^2^ 0,47. 0,53/ (0,05)^2^ x 2 = 766; une marge estimative d´environ 4% a été ajoutée à la taille de l'échantillon pour pallier le taux de non-réponses. Ce qui nous a permis d´obtenir un échantillon de 800 ménages.

### Variables

La variable dépendante était l´utilisation des services de santé (oui/non): elle était définie comme étant le recours aux soins aux services de santé (public ou privé) d´un membre du ménage lors du dernier épisode de la maladie survenue dans le ménage durant les douze derniers mois [5]. En ce qui concerne les variables indépendantes, elles étaient classées en plusieurs catégories. Celles-ci ont été tirées du modèle adapté d´Anderson, 1995, du modèle Health belief et de King [6-8]. Parmi lesquelles, nous avons: les facteurs prédisposants: sexe (masculin/féminin), statut marital (célibataire, en couple, marié, veuf ou séparé), religieux (oui/non), taille du ménage (1-2 personnes; 3-5 personnes; > 5 personnes). Les facteurs facilitants: revenu, affiliation à une assurance santé (oui/non), affiliation à une mutuelle de santé (oui/non), avoir reçu la visite d´un relais communautaire (oui/non), avoir reçu la visite d´un personnel de santé (oui/non). Les besoins pour soins: souffrir d´une maladie chronique (oui/non). Le système de soins et de santé: perception du délai d´attente au service de santé (court ≤ 15 minutes/long>15 minutes), l'accueil du personnel de santé, (bon/mauvais), la qualité des soins offerts (bonne/mauvaise), la disponibilité des médicaments (disponible/non disponible), la perception du coût des soins (faible/élevé). L´environnement externe: la distance par rapport au service de santé le plus proche (≤ 5km/> 5km).

### Saisie et analyse des données

Les données ont été collectées à l´aide de l´application Kobo Collect version 1.30 à partir des tablettes. L´analyse des données a été réalisée avec le programme R version 4.2.0. Les variables qualitatives ont été exprimées en effectif et pourcentage et les variables quantitatives en termes de moyenne, écart-type et extrêmes. Le taux d´utilisation des services de santé a été calculé et exprimé en pourcentage. En analyse bivariée, chaque variable indépendante a été croisée avec la variable dépendante. Les odds ratio bruts avec leurs intervalles de confiance à 95% et les p-values ont été calculés pour avoir la force de l´association. Les variables significatives en analyse bivariée ont été intégrées dans le modèle multivarié. Le seuil de significativité pour l´intégration dans le modèle de régression logistique binaire était fixé à 0,05. L´adéquation du modèle final a été vérifié par le test de Hosmer-Lemeshow.

### Considérations éthiques

Une autorisation de recherche a été obtenue auprès du responsable du District Sanitaire de Moungali. Avant l'entretien, chaque participant à l´étude a été clairement informé de la démarche de la recherche, de l´anonymat total dans la collecte de l'information. Un consentement libre et éclairé a été obtenu auprès de chaque participant ayant participé à l´étude.

## Résultats

### Analyse descriptive des variables indépendantes de notre échantillon

La population d´étude était constituée de 800 chefs de ménages, parmi lesquels il y avait 542 de sexe masculin (67.75%) et 258 de sexe féminin (32.25%). Le ratio femme/homme était de 2,1. L'âge moyen des chefs de ménages était de 43,15±13,47 ans. Les caractéristiques de notre échantillon en fonction de chaque variable indépendante sont décrites dans le Tableau 1 et le Tableau 2.

**Tableau 1:** caractéristiques de l'échantillon des chefs de ménage

Variables	Effectif N=800	Pourcentage %
**Sexe**		
Masculin	258	32,25
Féminin	542	67,75
**Age en années**		
Minimum; maximum	18 ; 83	
Moyenne ± Ecart-type	43,15±13,47	
Médiane [q1; q3]	41[32 ;52]	
**Tranche d'âge**		
18-30 ans	116	14,50
31-40 ans	223	27,88
41-50 ans	177	22,12
51-60 ans	118	14,75
61-70 ans	60	7,50
71-83 ans	28	3,50
Non spécifié	78	9,75
**Statut marital**		
Célibataire	214	26,75
En couple	366	45,75
Marié	138	17,25
Séparé	30	3,75
Veuf	52	6,5
**Taille ménage**		
1-2	115	14,37
3-5	390	48,75
> 5	293	36,63
Non spécifié	2	0,25
**Revenu**		
< Smig	437	54,62
> Smig	100	12,50
Non spécifié	263	32,88
**Assurance santé**		
Non	775	96,87
Oui	21	2,63
Non spécifié	4	0,5
**Mutuelle santé**;		
Non	757	94,63
Oui	40	5,00
Non spécifié	3	0,37
**Visite relais communautaire**		
Non	634	79,25
Oui	163	20,37
Non spécifié	3	0,38
**Visite agent de santé**		
Non	719	89,87
Oui	78	9,75
Non spécifié	3	0,38

**Tableau 2:** caractéristiques de l'échantillon des chefs de ménage

Variables	Effectif N=800	Pourcentage %
Maladie chronique		
Non	586	73,25
Oui	212	26,50
Non spécifié	2	0,25
**Délai d'attente**		
Long	436	54,50
Court	344	43,00
Non spécifié	20	2,5
**Accueil du personnel**		
Mauvais	239	29,87
Bon	554	69,25
Non spécifié	7	0,88
**Qualité des soins**		
Mauvaise	127	15,87
Bonne	665	83,13
Non spécifié	8	1,0
**Médicaments**		
Non disponible	459	57,37
Disponibilité	330	41,25
Non spécifié	11	1,38
**Distance**		
> 5km	256	32,00
< 5km	534	66,75
Non spécifié	10	1,25
**Coût des soins**		
Elevé	357	44,62
Faible	433	54,13
Non spécifié	10	1,25

Taux d´utilisation des services de santé: **le taux d'utilisation des services de santé des ménages dans le District Sanitaire de Moungali était de 27,62% IC à 95% [24,62-30,83]**.

### Analyse bivariée et multivariée des déterminants de l'utilisation des services de santé

A l'analyse bivariée, comme variables facilitants, l'utilisation des services de santé était significativement associée au revenu du ménage supérieur au SMIG (ORb=2,5 IC [1,57-3,98], p=0,0001) ainsi qu´au fait d´avoir reçu au moins une fois à domicile la visite d´un agent de santé durant l´année (ORb=2,21 IC 95% [1,37-3,57], p=0,0001). En ce qui concerne les variables classées dans le système de soins et de santé, parmi les variables significativement associées, il y avait: le délai d'attente court dans les services de santé (ORb=1,92 IC95% [1,40-2,64], p=0,0001); le bon accueil du personnel de santé (ORb=2 IC95% [1,39-2,9], p=0,0002); la bonne qualité des soins offerts (ORb=1,8 IC95% [1,12-2,9], p=0,015); le coût faible des prestations offertes (ORb=1,45 IC95% [1,06-1,99], p=0,022). Cependant, en analyse multivariée trois variables étaient associées à l´utilisation des services de santé: le délai d´attente court (≤ 15 minutes) dans les services de santé (ORa=1,90 IC95% [1,21-2,97] p=0,001); le revenu du ménage supérieur au smig (ORa=2,12 IC95% [1,30-3,48], p=0,003); et le faible coût des soins dans les structures de santé (ORa=1,73 IC95% [1,11-2,7], p=0,015). Ces résultats sont décrits dans le Tableau 3 et le Tableau 3.1. Le test de Hosmer-Lemeshow a montré une bonne adéquation du modèle aux données (p=0,268).

**Tableau 3:** analyse bivariée et multivariée des déterminants de l'utilisation des services de santé

Variables	Utilisation des Services de Santé		OR Brute	IC 95%	p	OR Ajust	IC 95%	p
	Oui	Non						
**Sexe**								
Féminin	161	381	1,39	[0,99-1,96]	0,057	-		
Masculin	60	198	1			-		
**Tranche d'âge**								
18-30 ans	37	79	1					
31-40 ans	63	160	0,84	[0,52-1,37]	0,485	-		
41-50 ans	45	132	0,73	[0,43-1,22]	0,228	-		
51-60 ans	30	88	0,73	[0,41-1,29]	0,274	-		
61-70 ans	15	45	0,71	[0,35-1,43]	0,343	-		
71-83 ans	11	17	1,38	[0,59-3,24]	0,458	-		
**Statut marital**								
En couple	82	284	0,62	[0,42-0,91]	0,013	-		
Mari	54	84	1,38	[0,88-2,16]	0,158	-		
Spar	4	26	0,33	[0,11-0,98]	0,047	-		
Veuf	13	39	0,72	[0,36-1,43]	0,342	-		
Célibataire	68	146	1			-		
**Taille ménage**								
1-2	42	73	1,56	[0,98-2,47]	0,058	-		
3-5	98	292	0,9	[0,64-1,28]	0,588	-		
> 5	79	214	1					
**Revenu**								
> Smig	39	61	**2,5**	[1,57-3,98]	0,0001	**2,12**	[1,30-3,48]	0,003
< Smig	89	348	1			1		
**Assurance santé**								
Oui	7	14	1,32	[0,53-3,31]	0,555	-		
Non	213	562	1			-		
**Mutuelle Santé**								
Oui	7	33	0,55	[0,24-1,26]	0,157	-		
Non	211	546	1			-		
**Visite relais communautaire**								
Oui	39	124	0,79	[0,53-1,17]	0,240	-		
Non	181	453	1			-		
**Visite agent de santé**								
Oui	34	44	2,21	[1,37-3,57]	0,001	1,64	[0,61-4,43]	0,325
Non	186	533	1			1		

**Tableau 3.1:** analyse bivariée et multivariée des déterminants de l'utilisation des services de santé

Variables	Utilisation des Services de Santé		OR Brute	IC 95%	p	OR Ajust	IC 95%	p
	Oui	Non						
**Maladie chronique**								
Oui	53	159	0,83	[0,58-1,19]	0,307	-		
Non	168	418	1			-		
**Délai d'attente**								
Court	121	223	**1,92**	[1,4-2,64]	0,0001	**1,90**	[1,21-2,97]	0,001
Long	96	340	1			1		
**Accueil personnel**								
Bon	176	378	**2**	[1,39-2,9]	0,0002	1,67	[0,89-3,11]	0,108
Mauvais	45	194	1			1		
**Qualité des soins**								
Bonne	197	468	**1,8**	[1,12-2,9]	0,015	0,98	[0,46-2,09]	0,966
Mauvaise	24	103	1			1		
**Médicaments**								
Disponibilité	94	236	1,06	[0,78-1,46]	0,699	-		
Non disponible	125	334	1			-		
**Distance**								
≤ 5km	153	381	1,13	[0,81-1,589]	0,467	-		
> 5km	67	189	1			-		
**Coût des soins**								
Faible	135	298	**1,45**	[1,06-1,99]	0,022	**1,73**	[1,11-2,7]	0,015
Elevé	85	272	1			1		

## Discussion

Dans notre étude, le taux d'utilisation des services de santé est de 27,62%. Les déterminants significativement associés à l´utilisation des services de santé sont: avoir un revenu supérieur au SMIG, un délai d'attente court dans les structures de soins et services de santé; ainsi que le coût faible des prestations dans les structures de santé.

### Taux d'utilisation des services de santé

Le taux d'utilisation des services de santé dans notre étude est de 27,62% IC à 95% [24,62-30,83]. Ce taux représente une utilisation des services de santé en moyenne de 0,3 nouveaux cas par habitant et par an. Ceci signifie qu´un habitant utilise les services de santé moins d´une fois tous le trois (3) ans. Notre taux est inférieur à celui rapporté par Simaga *et al*. dans une étude menée dans la commune de Bamako, soit 36,9% [9]. De plus, il est également inférieur à celui rapporté à Abidjan selon les données statistiques, soit 38,49%. On note aussi, 0,4 de consultation en moyenne par personne et par an pour Abidjan [10]. Notre taux est légèrement supérieur à celui du niveau national selon l´ECOM 2011 (enquête congolaise auprès des ménages pour l'évaluation de la pauvreté), soit 24% ou 0,24 consultations/habitant et par an. Selon le rapport de la revue du secteur de la santé 2018, en se référant de l´ECOM 2011, un congolais utilise les services de santé une fois tous les quatre (4) ans [11]. Nous constatons que l'ensemble de ces taux d´utilisation des services de santé des données provenant des enquêtes et études au niveau national comme ailleurs sont inférieurs à 50%. Ces taux sont loin des normes recommandées par l'OMS qui stipulent une utilisation minimum des services de Santé d´au moins une fois par an par habitant. De plus, ils restent assez éloignés des cibles définies par l´OMS pour atteindre la couverture sanitaire universelle d´ici 2030 [12-14]. Ces chiffres montrent tout simplement que plusieurs défis restent à relever dans le système de santé en République du Congo en vue de s´aligner et d´atteindre les différentes cibles pour la couverture sanitaire universelle d´ici 2030. Car l'utilisation des services de santé par les ménages est un déterminant majeur pour atteindre les différentes cibles.

### Déterminants de l´utilisation des services de santé

L'analyse multivariée montre que le revenu du ménage supérieur au SMIG, le délai d´attente court, le coût faible des prestations sont significativement associés à l'utilisation des services de santé.

### Revenu du ménage

En ce qui concerne le revenu du ménage supérieur au SMIG, ORA=2,12 IC95% [1,30-3,48], p =0,003, une enquête menée au Burkina Faso montre qu'un revenu faible pouvait être une contrainte à la consommation des soins. Ceci montre que le revenu du ménage joue un rôle important dans l'utilisation des services de santé. Nos résultats corroborent avec ceux de l´étude du Burkina Faso réalisée par Haddad *et al*. [15]. Plusieurs études ont déjà mis en relation l'utilisation des services de santé et le revenu des ménages [16]. Les données disponibles montrent que les personnes pauvres ont moins accès aux services de santé que les personnes à revenu élevé [17,18]. Ces disparités sont d´autant plus observées dans les pays en voie de développement [19,20].

### Coût des soins

Les coûts des soins jugés faibles par les ménages étaient également associés à l'utilisation des services de santé, ORA=1,73 IC95% [1,11-2,7], p=0,015. Le coût de soins peut s'avérer être un obstacle majeur de l'accès aux soins ou l´utilisation des services de santé. Cette observation a été faite dans une étude comportementale menée au Nigeria par Akeju *et al*. [21]. L'orientation du système de santé vers de mécanismes de protection financière durable, notamment les mécanismes de prépaiement, d'assurance et mutuelle permettra de réduire la barrière liée aux coûts de soins et garantir la couverture sanitaire universelle [22-24]. En République du Congo, le PNDS (Programme National de Développement Sanitaire) 2018-2022 fait état de l'opérationnalisation de l'assurance maladie et de mécanismes de prépaiement visant à s'orienter vers la couverture sanitaire universelle. Plusieurs recommandations institutionnelles mettent en avant le financement solidaire des dépenses de santé par la mise en place de l'assurance maladie universelle [25]. Ces mécanismes de partage de risque permettront d´assurer l'accessibilité financière à tous aux services de santé et garantir l'équité.

### Délai d´attente

Notre étude montre qu'un délai d'attente court était significativement associé à l'utilisation des services de santé, OR=1,90 IC95% [1,21-2,97], p=0,001. Le temps d´attente court était défini comme étant inférieur ou égal à 15 minutes et long comme étant supérieur à 15 minutes. Une étude réalisée en République Démocratique du Congo (RDC) par Mulinganya *et al*. rapporte qu’un temps d´attente prolongé était un facteur d´insatisfaction des patients avec pour conséquence la diminution de la fréquentation des services de santé [26]. Alors que la perception d'un temps d'attente court était associée à l'utilisation des services de santé d'après Thompson *et al*. [27]. Par ailleurs, le bon accueil du personnel et la bonne qualité des soins ont été significativement associés à l'utilisation des services de santé en analyse bivariée. Cependant ces variables n'ont pas été significatives en analyse multivariée. Toutefois, le bon accueil du personnel ou la bonne qualité de soins ont une bonne influence sur l'acceptation des services et la satisfaction des patients selon plusieurs études [28,29]. Beaucoup études ont mis en évidence la qualité de l'accueil du personnel.

### Limites de l´étude

Notre étude comporte certaines limites en lien avec l'entretien des ménages. Bien que les enquêteurs soient expérimentés, toutefois nous pensons qu´un biais de mémoire pourrait exister chez les ménages entretenus. Certains chefs de ménages ne pourraient se rappeler avec une précision totale de tout ce qui s'est passé dans le ménage rétrospectivement en lien avec les variables de l'étude. Car les informations demandées s'étant déroulées dans le passé ou durant les douze (12) derniers mois à partir du moment de l'enquête. C'est dans cette mesure que pour avoir toutes les réponses à notre formulaire d'enquête, ceux-ci étaient accompagnés de leurs conjoints ou conjointes ou d'autres membres selon les spécificités de chaque ménage. Cette approche a permis de réduire significativement ce biais de mémoire.

## Conclusion

Le taux d'utilisation des services de santé reste faible et assez éloigné des normes attendues par l'OMS. Le revenu du ménage, le coût des soins ainsi que le délai d'attente étant significativement associé à l'utilisation des services de santé, il est impératif de mettre en place des régimes d´assurance, de solidarité nationale ou les mutuelles. Ceci dans le but de limiter ou minimiser le paiement direct et à l'acte pour engendrer plus d'équité en santé et s'orienter vers la couverture sanitaire universelle. Ces actions nécessitent une intervention multisectorielle pour un meilleur impact.

### Etat des connaissances sur le sujet

- Une moitié de la population mondiale n'a pas accès aux services de santé essentiels;
- L'accès à tous aux services de santé est essentiel pour améliorer le bien-être de la population d´un pays;
- L'Afrique est le continent où la situation sanitaire et sociale est la plus préoccupante.

### Contribution de notre étude à la connaissance

- Le taux d´utilisation des services de santé des ménages dans le District Sanitaire de Moungali est de 27,62% (0,28 nouveaux cas/hab/an);
- Un habitant utilise les services de santé moins d´une fois tous les trois (3) ans au District Sanitaire de Moungali;
- Le délai d´attente court dans les services de santé (inférieur ou égal à 15 minutes), un revenu mensuel supérieur au SMIG et le coût des soins faible sont associés à l´utilisation des services de santé.
